# Magnetic fluorescent carbon dots synthesized via one-pot approach for tumor photothermal therapy

**DOI:** 10.1016/j.isci.2025.114366

**Published:** 2025-12-11

**Authors:** Yunyang Zhao, Jie Liu, Deyang Liu, Qiufang Gong, Zaisheng Wu, Songnan Qu, Chao Liang

**Affiliations:** 1 Institute for Advanced Research, Affiliated Cixi Hospital, Wenzhou Medical University, Wenzhou, Zhejiang 315300, China; 2 Scientific Research Center, Cixi Biomedical Research Institute, Wenzhou Medical University, Wenzhou, Zhejiang 315300, China; 3 Clinical Laboratory Center, The Second Affiliated Hospital of Wenzhou Medical University, Wenzhou, Zhejiang 325027, China; 4 School of Information Engineering, Anhui University of Chinese Medicine, Hefei, Anhui 230012, China; 5 School of Laboratory Medicine and Life Sciences, Wenzhou Medical University, Wenzhou, Zhejiang 325035, China; 6 Institute of Applied Physics and Materials Engineering, University of Macau, Taipa, Macau SAR 999078, China

**Keywords:** nanoparticles, biological sciences, bioengineering, Cancer, materials science, biomaterials

## Abstract

Carbon dots (CDs) exhibit low toxicity and excellent biocompatibility, endowing them with great potential in tumor photothermal therapy (PTT). However, existing magnetic-targeting CD composites are plagued by complex synthesis processes and fluorescence quenching issues. In this work, core-shell structured CDs@Fe_3_O_4_ is synthesized via a one-step microwave hydrothermal method. It integrates magnetic responsiveness, solid-state fluorescence, and photothermal conversion capabilities—with polyethyleneimine (PEI) acting as a bifunctional linker to bridge Fe_3_O_4_ nanoparticles and CDs, forming a puffy cluster structure that mitigates aggregation-induced fluorescence quenching (AIQ) for efficient solid-state emission. Notably, its magnetic property enables enrichment at target sites under external magnetic fields, while its photothermal effect efficiently converts laser energy into heat for tumor ablation. These features highlight CDs@Fe_3_O_4_ as a promising magnetically targeted photothermal agent for precision cancer therapy.

## Highlights

• A one-pot microwave hydrothermal method synthesizes core-shell CDs@Fe_3_O_4_
• Integrates magnetic fluorescent properties; PEI/FRET prevents fluorescence quenching
• Magnetic field enhances tumor-site material enrichment for precise tumor PTT ablation
• Multi-functional CDs@Fe_3_O_4_: good biocompatibility, rapid metabolism, and high safety

## Graphical abstract

**Figure:**
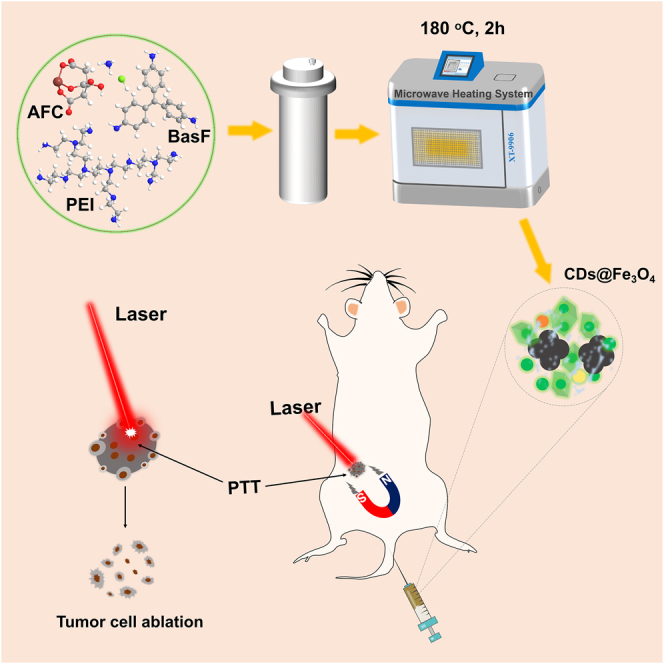

## Introduction

Carbon dots (CDs), a novel fluorescent carbon nanomaterial, outperform traditional quantum dots with low toxicity, environmental friendliness, excellent biocompatibility, tunable photoluminescence (PL) wavelengths, and high photochemical stability.^1^^,^^2^ These features make CDs highly promising for biomedical applications, especially photothermal therapy (PTT).^3^^,^^4^

Malignant tumors pose a significant threat to human life and health. Owing to their photothermal effect and tumor-targeting design, CDs can be applied in tumor PTT.^3^^,^^5^^,^^6^ Many studies achieve tumor targeting via intratumoral injection or the enhanced permeability and retention effect,^3^^,^^7^^,^^8^ but these methods often result in weak tumor targeting of CDs. Magnetic targeting-enhanced PTT has become a vital technique in medical applications, particularly for improving the efficacy and safety of targeted disease treatment. Thus, magnetic targeting is crucial for the precise PTT of CDs. However, synthesizing magnetic composites with CDs has been challenging due to the aggregation-induced fluorescence quenching (AIQ) of CD clusters and the strong visible-light absorption of Fe_3_O_4_. Typically, such magnetic fluorescent composites require two-step or multi-step processes to form multi-layered core@shell structures.^9^^,^^10^

**Scheme 1:**
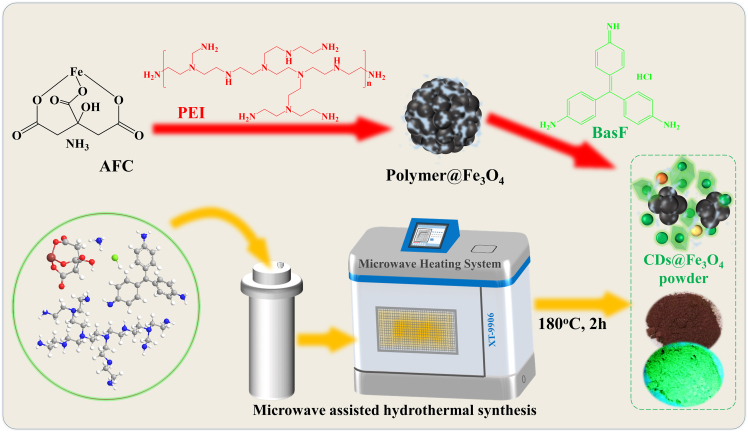
The synthesis process of CDs@Fe_3_O_4_

In this study, we report a simple one-pot microwave-assisted hydrothermal method to synthesize core-shell structured magnetic fluorescent nanomaterials (CDs@Fe_3_O_4_) as illustrated in Scheme 1. Under microwave heating, ammonium ferric citrate (AFC) is converted into magnetic Fe_3_O_4_ (Polymer@Fe_3_O_4_) with the aid of polyethyleneimine (PEI). Simultaneously, basic fuchsin (BasF) is carbonized into green emissive CDs on the Fe_3_O_4_ surface through PEI-mediated crosslinking. This one-pot synthesized CDs@Fe_3_O_4_ exhibits rare solid-state green emission. Finally, we applied the solid-state emissive magnetic CDs@Fe_3_O_4_ in tumor PTT.

## Results

### Magnetic and optical properties

**Figure 1:**
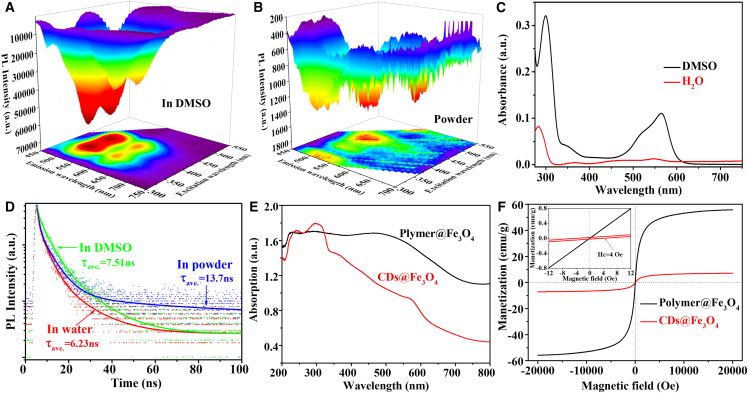
The optical and magnetic properties of Polymer@Fe_3_O_4_ and CDs@Fe_3_O_4_ The EEM of CDs@Fe_3_O_4_ in DMSO (A) and powder state (B). (C) Absorption spectra of CDs@Fe_3_O_4_ in water and DMSO. (D) TRPL decay curve of CDs@Fe_3_O_4_ in water, DMSO, and powder state. (E) Absorption spectra of Polymer@Fe_3_O_4_ and CDs@Fe_3_O_4_ powder. (F) The room temperature magnetization (M-H) curves of Polymer@Fe_3_O_4_ and CDs@Fe_3_O_4_. The insert showed the enlarged M-H curves.

Solid-state emissive CDs@Fe_3_O_4_ was first synthesized via a one-pot microwave-hydrothermal method and subsequently purified by magnetic separation, ensuring the retention of its magnetic properties (Scheme 1; Figure S1). Figures 1A, 1B, and S2 show the excitation-emission maps (EEMs) of CDs@Fe_3_O_4_ in aqueous, dimethyl sulfoxide (DMSO) suspensions, and as a powder. In water, CDs@Fe_3_O_4_ nanoparticles emit green light at 520 nm, independent of the excitation wavelength. However, in DMSO solutions and the powder state, they exhibit both green and red emissions. This occurs because the electron-acceptor S=O groups in DMSO alter the electron cloud density on the CD surface, narrowing the optical band gap and enhancing absorption at 565 nm (Figure 1C) and emission around 610 nm.^11^ As measured, the photoluminescence quantum yield of CDs@Fe_3_O_4_ is 17.34% in powder (due to Fe_3_O_4_ matrix inhibiting CD π-π stacking for effective fluorescence) and 5.01% in aqueous solution (owing to CD detachment causing Fe_3_O_4_ absorption enhancement and CD fluorescence quenching), as we measured.

The CDs@Fe_3_O_4_ exhibits red emission in the solid state via fluorescence resonance energy transfer (FRET).^12^^,^^13^ The moderate spacing between emissive CDs in the nanocomposites suppresses AIQ. As shown in the time-resolved PL (TRPL) decay curves in Figure 1D, FRET from the green to red emission centers results in a longer red fluorescence lifetime for CDs@Fe_3_O_4_ powder compared to its aqueous form. In water, short-wavelength emissive crosslinked chain polymers in CDs@Fe_3_O_4_ dissolve, reducing FRET between fluorophores, which explains the excitation-independent PL property seen in Figure S2.

The solid-state emission of CDs@Fe_3_O_4_ is further elucidated by the powder absorption spectra in Figure 1E. We defined R_a_ as the ratio of absorption at 300 nm to that at 800 nm (R_a_ = Abs_(λ=300 nm)_/Abs_(λ=800 nm)_), which approximates the absorption ratio of CDs to Polymer@Fe_3_O_4_. Calculations show that CDs@Fe_3_O_4_ exhibited a R_a_ value of 4.1, significantly higher than the 1.5 of Polymer@Fe_3_O_4_. This indicates that Fe_3_O_4_ neither blocks CD absorption nor quenches its emission in the powder state, enabling the solid-state emission of CDs@Fe_3_O_4_.

To investigate the origin of magnetic fluorescent properties during CDs@Fe_3_O_4_ synthesis, control samples with/without PEI and BasF were prepared under identical conditions. Fluorescent photographs (Figure S3) revealed that iron source alone (AFC) failed to generate magnetic or fluorescent products. The addition of PEI yielded magnetic Polymer@Fe_3_O_4_ particles, indicating its role in promoting Fe_3_O_4_ formation but not carbonization into CDs. Conversely, the combination of PEI and BasF resulted in CDs@Fe_3_O_4_ with dual magnetic and PL properties. UV-vis analysis (Figure S4) suggested four key roles of PEI: (1) stabilizing Fe_3_O_4_ formation, (2) promoting CD synthesis, (3) crosslinking Fe_3_O_4_ and CDs, and (4) spacing emissive centers to mitigate AIQ.

**Figure 2:**
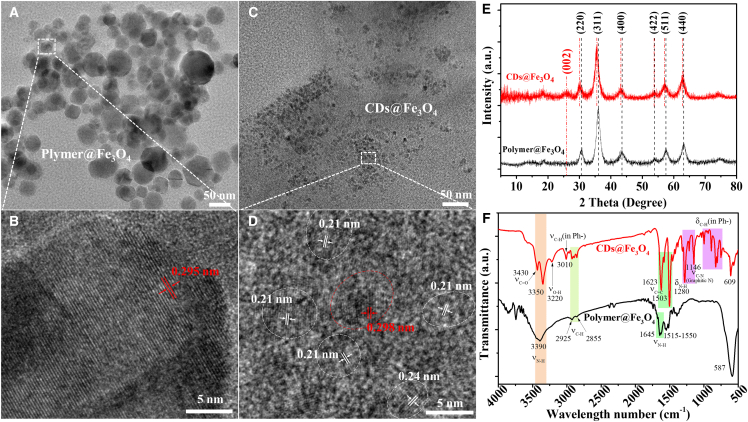
The crystal and chemical structure of Polymer@Fe_3_O_4_ and CDs@Fe_3_O_4_ The TEM and HRTEM of Polymer@Fe_3_O_4_ (A and B) and CDs@Fe_3_O_4_ (C and D). The XRD pattern (E) and FTIR spectra (F) of Polymer@ Fe_3_O_4_ and CDs@Fe_3_O_4_. Scale bars: (A and C) 50 nm; (B and D) 5 nm.

Magnetic properties were characterized by vibrating sample magnetometry (Figures 1F and S5). Saturation magnetization (M_s_) values of Polymer@Fe_3_O_4_ and CDs@Fe_3_O_4_ were 55.79 and 7.12 emu/g, respectively. The reduced M_s_ in CDs@Fe_3_O_4_ correlates with its smaller particle size (transmission electron microscopy [TEM], Figures 2A–2D) and higher non-magnetic CD content (R_a_ analysis, Figure 1E).^14^^,^^15^ The inset of Figure 1F shows a slight hysteresis loop (coercivity H_c_ = 4 Oe) in CDs@Fe_3_O_4_, indicating that CDs act as magnetic domain walls, impeding magnetization reversibility. This contrasts with the superparamagnetic behavior of Polymer@Fe_3_O_4_ (zero hysteresis). These results confirm that CDs modulate Fe_3_O_4_ size and endow the nanocomposite with paramagnetic characteristics.

### The crystal and chemical structure

TEM and high-resolution TEM (HRTEM) characterized the microstructure of Polymer@Fe_3_O_4_ and CDs@Fe_3_O_4_. TEM images (Figures 2A and 2C) revealed that Polymer@ Fe_3_O_4_ formed large nanoclusters (>20 nm), whereas CDs@Fe_3_O_4_ nanoparticles exhibited a significantly smaller diameter (<10 nm), indicating that BasF carbonized into CDs, inhibiting Fe_3_O_4_ aggregation. The size distribution histograms from TEM were provided in Figure S6, and the trend of this distribution is consistent with the dynamic light scattering results (Figure S7). HRTEM imaging (Figures 2B and 2D) showed that Polymer @Fe_3_O_4_ had a polycrystalline Fe_3_O_4_ structure, while CDs@Fe_3_O_4_ displayed fluorescent crystalline CDs encapsulating magnetic Fe_3_O_4_ cores. Lattice fringes with spacings of 0.21 and 0.24 nm, corresponding to graphene (100) and (1120) planes, confirmed the graphitic core of CDs in CDs@Fe_3_O_4_.^16^^,^^17^^,^^18^ The 0.30 nm lattice spacing matched the cubic Fe_3_O_4_ (220) plane, aligning with the X-ray diffraction (XRD) diffraction peak at 2θ = 31° (Figure 2E).^19^^,^^20^ XRD further elucidated the crystal structures. Diffraction peaks at 31°, 36°, 43.5°, 54°, 58°, and 63.5° corresponded to the (220), (311), (400), (422), (511), and (440) planes of Fe_3_O_4_, validating the paramagnetic nature of CDs@Fe_3_O_4_.^19^^,^^21^ A new peak at 26° in CDs@ Fe_3_O_4_ indicated the presence of graphitic CDs. Notably, the diffraction peaks of CDs@ Fe_3_O_4_ shifted toward lower angles, reflecting an increase in lattice spacing from 0.295 nm (Polymer@Fe_3_O_4_) to 0.298 nm (CDs@Fe_3_O_4_) as observed by HRTEM. This lattice expansion, consistent with Bragg’s law, confirmed the stable interfacial interaction between Fe_3_O_4_ and CDs.

The surface functional group of Polymer@Fe_3_O_4_ and CDs@Fe_3_O_4_ was characterized by Fourier transform infrared (FTIR) spectroscopy (Figure 2F). Compared with Polymer@Fe_3_O_4_, CDs@Fe_3_O_4_ exhibited peaks at 750–1,000, 3,010, and 1,623/1,503 cm^−1^, corresponding to the bending and stretching vibrations of C–H and C=C in aromatic structures, indicating the presence of sp^2^ C structures and confirming the successful synthesis of CDs.^22^ Additionally, a new C–N stretching vibration peak at 1,146 cm^−1^ suggested that CDs were connected to Fe_3_O_4_ in CDs@Fe_3_O_4_ via C–N bonds from PEI, verifying the role of PEI as a crosslinking agent.

**Figure 3:**
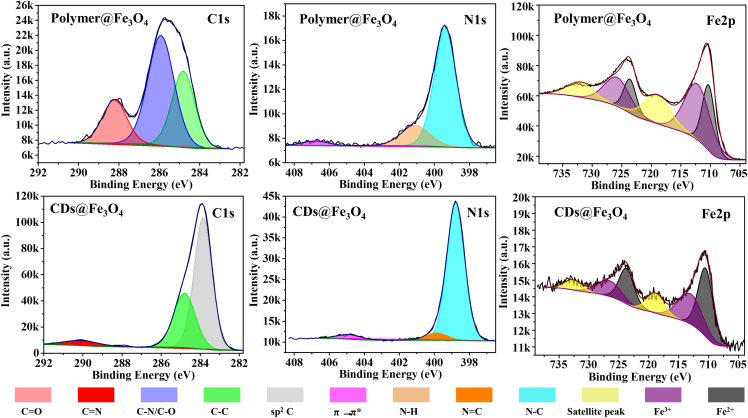
The high-resolution XPS of C 1s, N1s and Fe 2p spectra of Polymer@Fe_3_O_4_ and CDs@Fe_3_O_4_

To further elucidate the chemical structure of CDs@Fe_3_O_4_, X-ray photoelectron spectroscopy (XPS) was performed (Figures 3 and S9; Table S1). High-resolution C 1s spectra revealed a significant proportion of conjugated sp^2^ C in CDs@Fe_3_O_4_, indicating the formation of graphitic structures in CDs. The notable C–N content (10.65%) in the N 1s spectra confirmed PEI-mediated crosslinking between Fe_3_O_4_ and CDs. Analysis of Fe 2p spectra showed differing Fe^3+^ (10.88%) and Fe^2+^ (6.21%) ratios in Polymer@Fe_3_O_4_, suggesting incomplete conversion of AFC to Fe_3_O_4_. In contrast, CDs@Fe_3_O_4_ exhibited nearly equivalent Fe^3+^ (0.43%) and Fe^2+^ (0.56%) contents, consistent with stoichiometric Fe_3_O_4_ formation. The slightly elevated Fe^2+^ content in CDs@Fe_3_O_4_ may facilitate interactions with PEI, further supporting the crosslinking role of PEI. Elemental analysis revealed that Polymer@Fe_3_O_4_ contained ∼70% Fe and O, indicative of high Fe_3_O_4_ loading, whereas CDs@Fe_3_O_4_ comprised over 80% C, reflecting a high CD content. The higher C/Fe ratio in CDs@Fe_3_O_4_, consistent with powder absorption spectroscopy results (Figure 1E), confirms that Fe_3_O_4_ in CDs@Fe_3_O_4_ not only preserves CD emission but also acts as a matrix enabling strong solid-state emission via AIQ prevention.

### Fe_3_O_4_ expanded CD packing for solid-state luminescence

The PL mechanism of CDs is complex due to their intricate chemical structures. Most CDs lack solid-state PL properties because of the AIQ effect from π-π stacking. Typically, CDs/Fe_3_O_4_ nanocomposites struggle to exhibit such properties due to the strong visible light absorption of Fe_3_O_4_. In this study, we analyzed the CDs@Fe_3_O_4_ structure. BasF was condensed into graphene structure under microwave heating to form the graphitic core of CDs (Figure 2D), and PEI assisted the surface functional group generation of CDs and connected with Fe_3_O_4_, maintaining a distance between them to form a fluffy cluster. This spacing reduced Fe_3_O_4_-induced PL quenching of CDs, promoted FRET among CDs in CDs@Fe_3_O_4_, and inhibited the AIQ effect, enabling solid-state emission. Moreover, CDs influenced the magnetic properties of Fe_3_O_4_ in the composite. This reciprocal modulation of fluorescence and magnetism yielded unique magnetic CDs@Fe_3_O_4_ with solid-state emission capabilities.

### The photothermal effect and magnetic guidance

**Figure 4:**
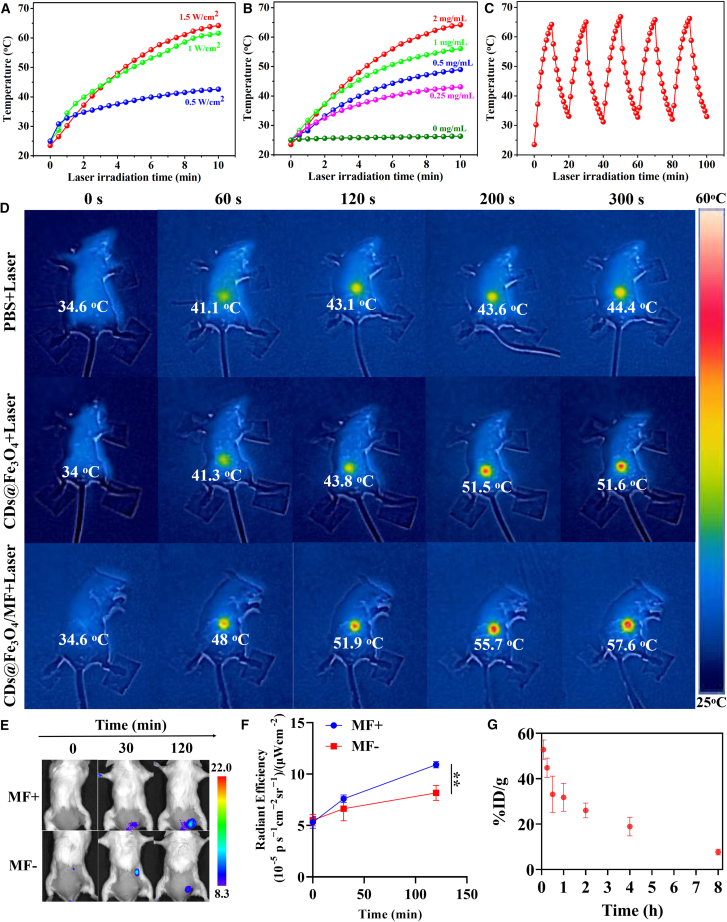
The photothermal effect, thermographic images, and biosafety evaluation of CDs@Fe_3_O_4_ The photothermal effect (temperature versus time, T-t) curves of CDs@Fe_3_O_4_. (A) T-t curves of CDs@Fe_3_O_4_ under different laser powers. (B) T-t curves with different concentrations of CDs@Fe_3_O_4_. (C) T-t curve with five cycles of laser ON/OFF. (D) The IR thermographic images with laser irradiation during PTT *in vivo*. (E) IVIS imaging of mice administered CDs@Fe_3_O_4_: without MF vs. different MF enrichment times. (F) The radiant efficiency *in vivo* of CDs@Fe_3_O_4_-administered mice from (E) IVIS imaging. (G) Blood circulation (%ID/g) of CDs@Fe_3_O_4_-administered mice as a function of time. Data are represented as mean ± SEM. Error bars were standard errors based on five mice in each group (*n* = 5 mice). Statistical significance was calculated by one-way ANOVA with Tukey post hoc test (∗∗*p* < 0.01, ∗∗∗∗*p* < 0.0001).

As shown in Figures 4A–4C, the photothermal effect of CDs@Fe_3_O_4_ was evaluated using a 655 nm laser at 1 W cm^−2^. Figure 4A demonstrates that under 655 nm laser irradiation for 10 min, the temperature of CDs@Fe_3_O_4_ aqueous solution increased from 42.6°C to 61.6°C and then 65.7°C at power densities of 0.5, 1.0, and 1.5 W cm^−2^, respectively, indicating that laser-induced temperature growth nearly saturates above 1 W cm^−2^. In Figure 4B, CDs@Fe_3_O_4_ solutions with concentrations of 0, 0.25, 0.5, 1, and 2 mg mL^−1^ reached temperatures of 26.3, 43.1, 49, 56.1, and 65.7°C, respectively. Figure 4C shows that CDs@Fe_3_O_4_ retained excellent photothermal stability over five laser irradiation cycles. These results highlight CDs@Fe_3_O_4_ as a promising photothermal agent for PTT applications.

Leveraging the exceptional photothermal performance and paramagnetism of CDs@Fe_3_O_4_, we explored its feasibility for tumor PTT via intravenous injection in 4T1-tumor-bearing mice. Twelve mice were randomized into four groups: (1) PBS + Laser, received phosphate-buffered saline (PBS) followed by 655 nm laser irradiation (1 W cm^−2^, 5 min); (2) CDs@Fe_3_O_4_, injected with CDs@Fe_3_O_4_ (1 mg/mL, 100 μL) without laser exposure; (3) CDs@Fe_3_O_4_ + Laser, administered CDs@Fe_3_O_4_ with laser irradiation; and (4) CDs@Fe_3_O_4_/MF + Laser, injected with CDs@Fe_3_O_4_ under an external magnetic field (MF) during laser treatment to evaluate magnetic targeting. Infrared (IR) thermal imaging (Figure 4D) revealed that tumor temperatures reached 57.6°C within 5 min post-MF enrichment in the CDs@Fe_3_O_4_/MF + Laser group, compared to 51.6°C (CDs@Fe_3_O_4_ + Laser) and 44.4°C (PBS + Laser) under identical irradiation. These results underscore the enhanced PTT efficacy of MF-guided CDs@Fe_3_O_4_ accumulation. The temperature distribution near tumor suggested that the hyperthermic region induced by laser was localized exclusively to the tumor site and existed only transiently, thereby avoiding damage to normal tissues (Figure S10). The CDs@Fe_3_O_4_ enriched in tumor by MF was confirmed by the *in vivo* optical imaging from *in vivo* imaging system (IVIS) in Figures 4E and 4F. With the time, the fluorescent signal with MF guidance increased obviously, which suggests the effective MF enrichment of CDs@Fe_3_O_4_ in tumor. The Calcein-AM/PI dual-staining assay results also confirmed the enhanced PTT of the CDs@Fe_3_O_4_ under MF guidance (Figure S11). In addition, CDs@Fe_3_O_4_ also exhibit excellent stability in both colloidal dispersions and optical performance, which provides a reliable foundation for their PTT in tumor (Figure S12).

### Evaluation of biocompatibility and safety

Moreover, we investigated the pharmacokinetics of CDs@Fe_3_O_4_ in living mice after intravenous injection, and venous blood samples at various time points were collected to measure the CDs@Fe_3_O_4_ contents. The blood half-life time of 2 h indicated the rapid metabolism of CDs@Fe_3_O_4_ (Figure 4G). This suggests that the CDs@Fe_3_O_4_ was rapidly excreted through the kidney after intravenous administration, demonstrating their excellent biocompatibility and minimal or no biotoxicity. Hemolysis tests were performed to evaluate the biocompatibility of CDs@Fe_3_O_4_. The results in Figure S13 indicated that the CDs@Fe_3_O_4_ possesses reliable safety. In biochemical assays, mice without CDs@Fe_3_O_4_ administration served as controls, while experimental groups received CDs@Fe_3_O_4_ (Figure S14). Target biochemical markers (alanine aminotransferase, aspartate aminotransferase, blood urea nitrogen, and creatinine) were measured in both groups at 1 and 7 days post administration to assess dynamic changes induced by CDs@Fe_3_O_4_. Notably, no statistically significant differences in these markers were observed between experimental and control groups, indicating that CDs@Fe_3_O_4_ exerts no detrimental effects on liver or kidney function. These results strongly support the safety and potential of CDs@Fe_3_O_4_ for PTT of tumors.

### Tumor PTT efficacy assessment *in vivo*

**Figure 5:**
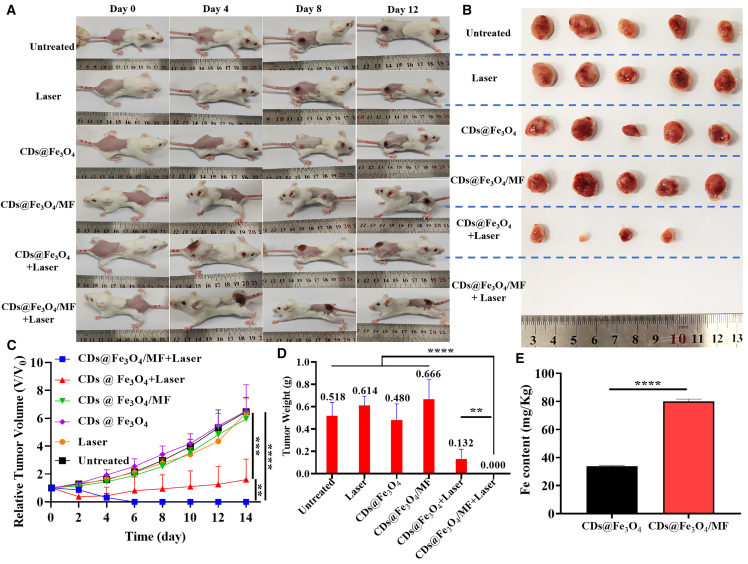
*In vivo* tumor PTT of CDs@Fe_3_O_4_ The photographs of 4T1 tumor-bearing mice (A) and tumor volume growth curves (C) in six groups within 14 days after PTT. The photographs of tumor tissue *in vitro* (B) and final tumor weight (D) in six groups on 14^th^ day after PTT. These six groups included the following: (1) Untreated, 4T1 tumor-bearing mice without any treatments like laser therapy and CDs@Fe_3_O_4_ administration; (2) Laser, 4T1 tumor-bearing mice with 655 nm laser irradiation (1 W cm^−2^, 5 min); (3) CDs@Fe_3_O_4_, only injected with CDs@Fe_3_O_4_ (1 mg/mL, 200 μL); (4) CDs@Fe_3_O_4_/MF, administered CDs@Fe_3_O_4_ with an external magnetic field (MF) enrichment; (5) CDs@Fe_3_O_4_ + Laser, administered CDs@Fe_3_O_4_ with laser irradiation; and (6) CDs@Fe_3_O_4_/MF + Laser, administered CDs@Fe_3_O_4_ with MF enrichment before laser treatment. (E) The Fe content in tumor tissues from ICP-OES results. Data are represented as mean ± SEM. Error bars were standard errors based on five mice in each group (*n* = 5 mice). Statistical significance was calculated by one-way ANOVA with Tukey post hoc test (∗∗*p* < 0.01, ∗∗∗∗*p* < 0.0001).

Given the favorable biosafety, good biocompatibility, and magnetically enrichable property of CDs@Fe_3_O_4_, their PTT efficacy against tumors was investigated and evaluated through these six groups as showed in Figures 5A–5D. Tumor volume analysis after 12 days (Figures 5A–5D) demonstrated significant growth suppression in “CDs@Fe_3_O_4_/MF + Laser” and “CDs@ Fe_3_O_4_ + Laser” groups. The tumors in the “CDs@Fe_3_O_4_/MF + Laser” group were completely ablated on the 6th day post PTT, which indicates that the MF enhanced the accumulation of CDs@Fe_3_O_4_ and thereby improved the therapeutic efficacy. In contrast, the first four groups lacked essential components (laser and photothermal agents) for PTT, leading to the continuous malignant progression of their tumors. These results underscore the synergistic requirement of a photothermal agent, laser, and magnetic guidance for effective tumor ablation.

Inductively coupled plasma optical emission spectrometry (ICP-OES) analysis of tumor Fe content (Figure 5E) quantified the magnetic enrichment effect. Tumor Fe concentrations reached 80.08 mg/kg in the CDs@Fe_3_O_4_/MF + Laser group, a 2.37-fold increase over the CDs@Fe_3_O_4_ + Laser group (33.8 mg/kg). This 237% enhancement in Fe accumulation directly correlates with the observed improvement in photothermal efficacy, confirming magnetic field-mediated enrichment of CDs@Fe_3_O_4_ in tumors. Scanning electron microscopy-energy dispersive spectroscopy mapping of freeze-dried tumors from the CDs@Fe_3_O_4_/MF + Laser group (Figure S16) confirmed colocalization of C, O, and Fe elements, verifying MF-driven tumor enrichment of CDs@Fe_3_O_4_.

Histopathological analysis via hematoxylin and eosin (H&E) staining was performed on major organs (heart, liver, spleen, lungs, and kidneys) and tumor tissues from all experimental groups (Figure S17). The CDs@Fe_3_O_4_/MF + Laser group exhibited no significant organ damage or inflammatory lesions, whereas varying degrees of pathological changes were observed in control groups: PBS + Laser and CDs@Fe_3_O_4_ groups: intact tumor cell morphology with tight arrangement, clear nuclei, and minimal necrosis; CDs@Fe_3_O_4_ + Laser group: focal tumor necrosis with preserved cellular architecture; and CDs@Fe_3_O_4_/MF + Laser group: widespread tumor cell lysis, nuclear pyknosis, and tissue disorganization indicative of complete ablation. Notably, control groups displayed inflammatory cell infiltration in the liver, disrupted splenic nodule architecture, and metastatic foci in lung tissues, confirming systemic tumor progression. In contrast, the CDs@Fe_3_O_4_/MF + Laser group showed no detectable lung metastases, underscoring the efficacy of magnetic-enhanced PTT. These findings validate that MF enrichment significantly enhances tumor ablation while mitigating off-target toxicity.

## Discussion

In summary, we present a facile microwave-assisted hydrothermal strategy for the one-pot synthesis of magneto-fluorescent CDs@Fe_3_O_4_ nanocomposites. PEI serves as a bifunctional linker, bridging Fe_3_O_4_ nanoparticles and CDs to form a puffy cluster structure that mitigates AIQ, enabling efficient solid-state emission. Leveraging their dual-modal properties, these nanocomposites exhibit MF-guided tumor accumulation, significantly enhancing PTT efficacy *in vivo*. Histopathological and ICP-mass spectrometry analyses confirm MF-mediated enrichment of CDs@Fe_3_O_4_ in tumors, leading to complete ablation. These results highlight CDs@Fe_3_O_4_ as a promising magnetically targeted photothermal agent for precision cancer therapy.

### Limitations of the study

Despite successfully synthesizing multifunctional nanomaterials integrating magnetic and solid-state luminescent properties, this work has several limitations. First, it lacks a systematic comparative analysis with other state-of-the-art nanomaterials, failing to highlight its unique advantages. Second, non-invasive magnetic enrichment remains challenging for deep-seated orthotopic tumors *in vivo*, restricting its clinical applicability. Third, the immune activation responses induced by the materials have not been investigated in depth, which hinders a comprehensive understanding of their biological interactions and potential immunotherapeutic synergies.

## Resource availability

### Lead contact

Requests for further information and resources should be directed to and will be fulfilled by the lead contact, Chao Liang (liangchao@wmu.edu.cn).

### Materials availability

CDs@Fe_3_O_4_ generated in this study will be made available on request, but we may require a payment and a completed materials transfer agreement if there is potential for commercial application.

### Data and code availability

• Raw data in formats such as TXT, Excel, Word, and RAR compressed files have been deposited at https://doi.org/10.5281/zenodo.17543960 and are publicly available as of the date of publication. Accession numbers are listed in the key resources table.
• This paper does not report original code.
• Any additional information required to reanalyze the data reported in this paper is available from the lead contact upon request.

## Acknowledgments

This work was financially supported by the Talent Scientific Research Start-up Fund of Wenzhou Medical University (grant no. 89222005), Zhejiang Province Natural Science Foundation (grant no. LQN25C100009), and 10.13039/100007834Ningbo Municipal Natural Science Foundation (grant no. 2023J395). The authors also gratefully acknowledge the Scientific Research Center of Wenzhou Medical University for providing instrumental support throughout this study.

## Author contributions

Conceptualization, Y.Z., S.Q., and C.L.; methodology, Y.Z. and C.L.; investigation and discussion, J.L., D.L., and Q.G.; writing – original draft, Y.Z.; writing – review and editing, Y.Z. and C.L.; funding acquisition, Y.Z. and C.L.; resources, C.L.; supervision, Z.W., S.Q., and C.L.

## Declaration of interests

The authors declare no competing interests.

## Declaration of generative AI and AI-assisted technologies in the writing process

During the preparation of this work, the authors used *Doubao* in order to polish the language or check grammatical errors to improve the clarity and fluency of the text. After using this tool or service, the authors reviewed and edited the content as needed and take full responsibility for the content of the publication.

## Star★Methods

### Key resources table

**Table:** 

REAGENT or RESOURCE	SOURCE	IDENTIFIER
**Chemicals, peptides, and recombinant proteins**		

Ammonium ferric citrate (AFC)	Aladdin Scientific	CAS#1185-57-5
Polyethylene imine (PEI)	Sigma-Aldrich	CAS#9002-98-6
Basic fuchsin (BasF)	Shanghai Macklin Biochemical Technology Co., Ltd	CAS#632-99-5

**Deposited data**		

All the raw data	Zenodo	https://doi.org/10.5281/zenodo.17543960

**Experimental models: Cell lines**		

4T1 Cell line	American Type Culture Collection (ATCC)	RRID: CVCL_0125

**Experimental models: Organisms/strains**		

Balb/c mice	Henan SCBC Biotechnology Co., LTD	IMSR_JAX:000651

**Software and algorithms**		

Origin 2016	Origin	Origin 2016
GraphPad Prism 9.3GraphPad Prism	GraphPad Prism	GraphPad Prism 9.3
ChemDraw 14	ChemDraw	ChemDraw 14

### Experimental model and study participant details

#### Animals, tumor models and ethics declarations

Female Balb/c mice aged 7 weeks with a body weight of 20–25 g per mouse were selected and placed in a standard laboratory animal housing environment (temperature: 22 ± 2 °C, relative humidity: 50 ± 10%, 12 h light/dark cycle) in Wenzhou Medical University. They were fed with standard rodent chow and sterile drinking water *ad libitum* for one week of acclimatization.

A 4T1 tumor mouse model was established in 8-week-old female Balb/c mice by subcutaneous injection of 4T1 cells. Specifically, Specifically, 5 × 105 4T1 cells in 100 μL were inoculated into both sides of the dorsal region of each mouse. Tumor growth was monitored by measuring the longest and shortest diameters using a vernier caliper, and tumor volume (V) was calculated with the formula V = 1/2 × a × b2, where a denoted the major axis and b represented the minor axis

All animal procedures were performed in accordance with the Guidelines for Care and Use of Laboratory Animals of Wenzhou Medical University and approved by the Animal Ethics Committee of Wenzhou Medical University (Certificate number: wydw2025-0026).

### Method details

#### The synthesis of Fe_3_O_4_

The synthesis of Fe_3_O_4_Typically, AFC (1.5 g) and PEI (1 mL) was dissolved into 10 mL ultrapure water. Then stirring for 5 min, this solution was sealed into a Teflon autoclave and transferred into the microwave oven (XT-9906, Closed intelligent microwave digestion instrument, Shanghai XTrust Analytical Instruments Co., LTD). After microwave hydrothermal reaction at 180 °C for 2 h, the magnetic suspension was obtained. This suspension was purified by magnetic separations for several and lyophilized to get Fe_3_O_4_ sample for reference.

#### The synthesis of CDs@Fe_3_O_4_

The synthesis of CDs@Fe_3_O_4_ is similar to the synthesis process of Fe_3_O_4_ except the addition of BasF in the reaction precursors. AFC (1.5 g), PEI (1 mL) and BasF (1 g) was dissolved into 10 mL ultrapure water to form a homogenous solution. This solution reacted in the XT-9906 microwave oven at 180 °C for 2 h, the magnetic fluorescent suspension was obtained. After purification with magnetic separations and freeze drying, the CDs@Fe_3_O_4_ powder were finally acquired.

#### Characterization

The excitation emission maps were also obtained on the FS5 fluorescence spectrophotometer (FS5, Edinburgh instruments Ltd., UK) by using a Xe lamp as an excitation source with the excitation wavelength step of 5 nm. The used holder was SC-20 sample module with cuvette holder under room temperature. Time-resolved PL decay curves were tested by the technique of Time Correlated Single Photon Counting (TCSPC) on the fluorescence spectrophotometer (FLS1000, Edinburgh instruments Ltd., UK) with a ps pulsed laser of EPL-375. UV ∼ vis absorption spectra were recorded on a spectrophotometer (UV-2600, Shimadzu Corporation, Japan). The M-H curves were investigated by vibrating specimen magnetometer (LakeShore7404, Lake Shore, USA). The TEM images were acquired by a Transmission Electron Microscope (JEM-F200, JEOL, Japan) operating at 200 kV. The magnetic samples were prepared by being dispersed in water/ethanol, then dropped on an opened double copper grid with carbon film and dried under an infrared baking lamp and closed it. FTIR spectra tested on Fourier Transform Infrared Spectrometer (NICOLET is20, Thermo, USA). XRD pattern was performed on X-ray diffractometer (Ultima IV, Rigaku Corporation, Japan). XPS spectra was carried out on an X-ray photoelectron spectroscopy (ESCALAB 250Xi, Thermo Fisher Scientific, USA) at 12.5 kV under the pressure of 8∗10^−10^ Pa. The binding energy C 1s = 284.60 eV is used as the energy standard to correct the charge. ICP-OES was performed on Thermo-ICPOES7200 (ThermoFisher, USA). SEM-EDS mapping was obtained on scanning electron microscope-energy dispersive spectrometer (SU8600, Hitachi High-Tech Corporation, Japan). *In vivo* imaging system (IVIS) was use the IVIS Spectrum (PerkinElmer, USA). Biochemical marker were tested on biochemical analyzer (AU480, Beckman Coulter, Inc., USA). The particle size distribution and zeta potential of the samples were characterized using a Zetasizer Nano ZS90 (Malvern Panalytical Ltd., Worcestershire, UK), a state-of-the-art dynamic light scattering (DLS) instrument widely recognized for its high precision in colloidal and nanoparticle analysis.

#### Photothermal effect measurement

Photothermal effect measurements were conducted using UT320A digital thermometers equipped with K-type thermocouples. Specifically, 1 mL of CDs@Fe_3_O_4_ aqueous solutions was transferred into quartz cuvettes. Subsequently, these cuvettes were exposed to a 655 nm laser at a power density of 1 W cm-^2^ for a duration of 10 min. Pure water served as the control group. The thermocouple probe of the digital thermometer was inserted into the tested solutions, and the temperature was recorded by the thermometer at 30-s intervals.

#### *In vivo* PTT

Once the tumor volume reached 100-200 mm^3^, the tumor-bearing Balb/c mice were randomly allocated into six groups (n = 5 mice per group):

1 Untreated group: Mice received an injection of phosphate-buffered saline (PBS) without laser treatment.
2 Laser group: Mice received an injection of phosphate-buffered saline (PBS) followed by irradiation with a 655 nm laser at a power density of 1 W cm^-2^ for 5 min.
3 CDs@Fe_3_O_4_ group: Mice were injected with 100 μL of CDs@Fe_3_O_4_ solution (1 mg/mL) without laser treatment.
4 CDs@Fe_3_O_4_/MF group: Mice were injected with CDs@Fe_3_O_4_ solution while an external magnetic field (MF) was applied on the tumor site without laser treatment.
5 CDs@Fe_3_O_4_+Laser group: Mice were administered CDs@Fe_3_O_4_ solution and then exposed to the 655 nm laser.
6 CDs@Fe_3_O_4_/MF + Laser group: Mice were injected with CDs@Fe_3_O_4_ solution while an external magnetic field (MF) was applied during laser irradiation to assess magnetic targeting capabilities.

Tumor site temperature changes were continuously monitored and recorded using an FLIR E5 Pro thermal imaging camera (FLIR Systems Inc.). All mice were anesthetized using Zoletil (Virbac, Carros, France) before each measurement procedure to ensure animal welfare and accurate data acquisition.

### Quantification and statistical analysis

All the nanomaterial characterization data were plotted using Origin 2016. All *in vivo* and *in vitro* biology-related data are presented as mean ± SEM, with the corresponding statistical analysis performed using GraphPad Prism 9.3. Error bars were standard errors based on five mice in each group (n = 5 mice). Statistical significance was calculated by one-way ANOVA with Tukey post hoc test (∗∗P < 0.01, ∗∗∗P < 0.001, ∗∗∗∗P < 0.0001). Chemical structures were drawn using ChemDraw 14. All of the statistical details of experiments can be found in the figure legends.
