# Supplementary material for: Magnetic fluorescent carbon dots synthesized via one-pot approach for tumor photothermal therapy
Source: iScience. 2025 Dec 11;29(1):114366. doi: 10.1016/j.isci.2025.114366 (PMC12796531; doi:10.1016/j.isci.2025.114366)
Supplement: Document S1. Figures S1–S17 and Table S1 [file mmc1.pdf]

iScience, Volume *29*

Supplemental information

Magnetic fluorescent carbon dots synthesized via one-pot approach for tumor photothermal therapy

Yunyang Zhao, Jie Liu, Deyang Liu, Qiufang Gong, Zaisheng Wu, Songnan Qu, and Chao Liang

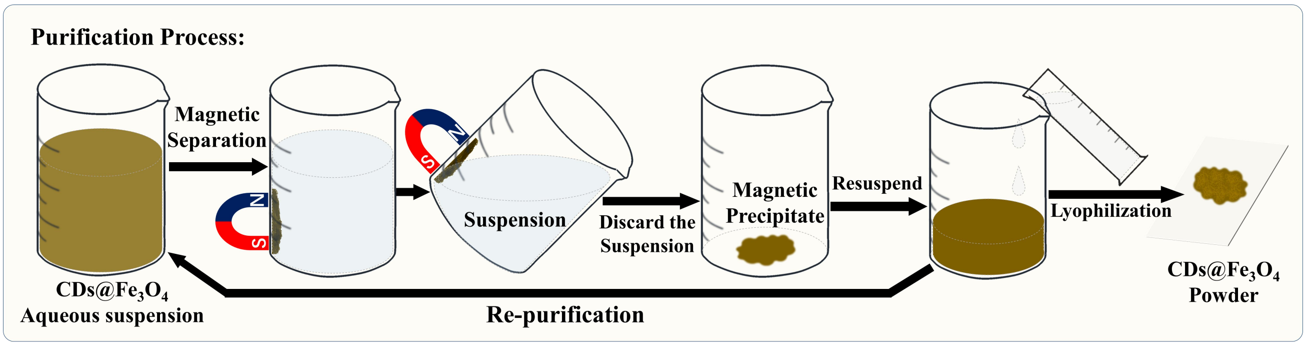

Figure S1. The purification process of magnetic nanomaterials. Take CDs@Fe_3O_4 as an example.

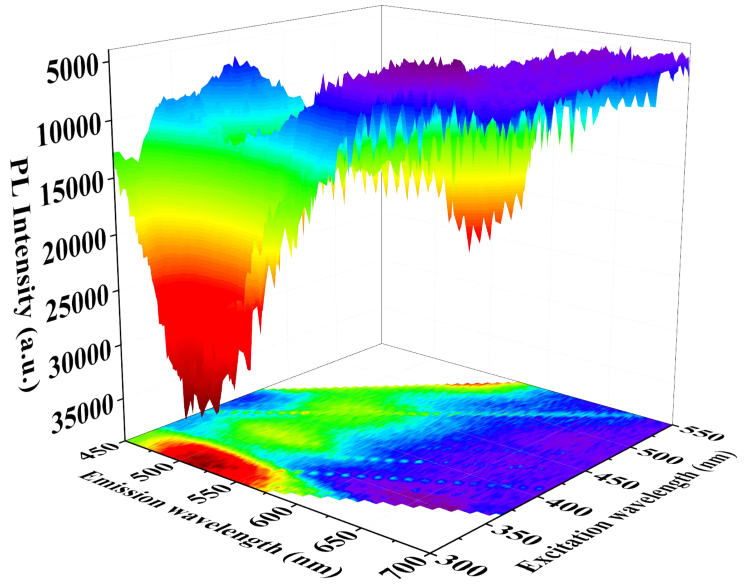

Figure S2. The EEM of $CDs@Fe_3O_4$ in water

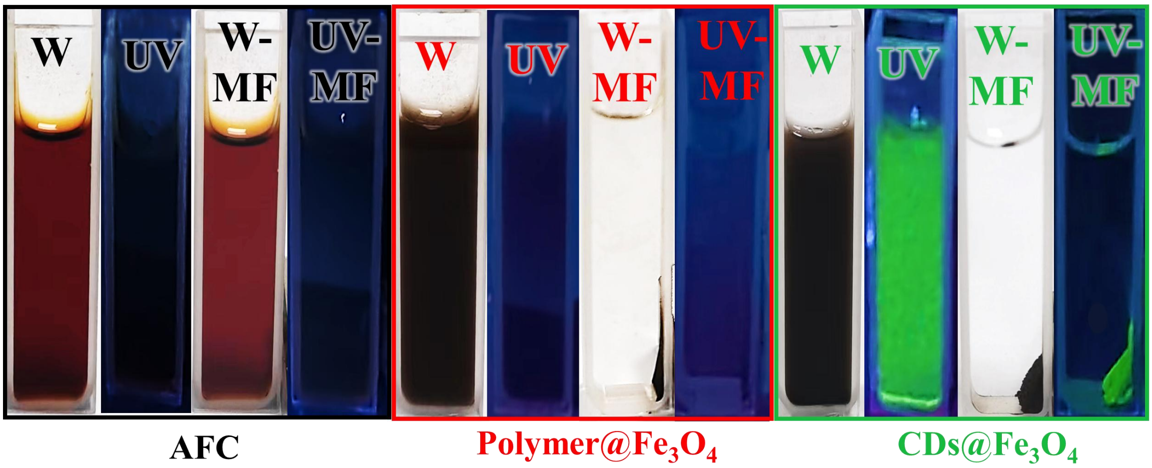

Figure S3. The photograph of AFC, Polymer@Fe_3O_4 and CDs@Fe_3O_4 under white light (W) and 365nm light (UV) without/with magnetic force (MF) on the right. "W" and "UV" means that the pictures were acquired under white and 365 nm UV light, respectively. "MF" means that the pictures were obtained under the influence of magnetic force on the right side.

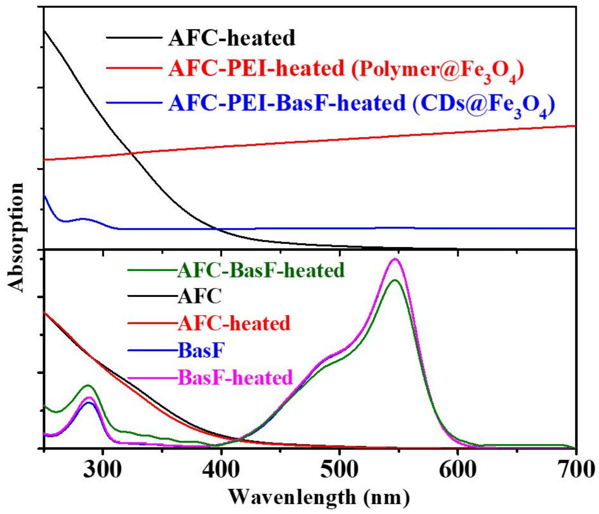

Figure S4. The UV~vis absorption of different samples aqueous suspension synthesized from different precursors. The reactant ratio is the same as that of Polymer@Fe_3O_4 and CDs@Fe_3O_4. The "heated" means that the reaction condition is microwave hydrothermal reaction at 180 °C for 2 hours, which is the same as that of Polymer@Fe_3O_4 and CDs@Fe_3O_4.

From the absorption spectra, we can find that AFC and AFC-heated have the nearly coincident absorption curve which confirms that AFC cannot transform into Fe_3O_4 without PEI. Similarly, the coincident absorption curve of BasF before and after microwave heating means BasF cannot be carbonized into CDs lonely under this reaction condition. And the green curve of "AFC-BasF-heated" is about the sum of that of "AFC-heated" and "BasF-heated", which indicates BasF cannot react with AFC without PEI addition. Above all, the role of PEI can be summarized: (1) to promote the formation of Fe_3O_4; (2) to promote the synthesis of CDs; (3) to connect the Fe_3O_4 and CDs; (4) to prevent the AIQ for solid-state emission

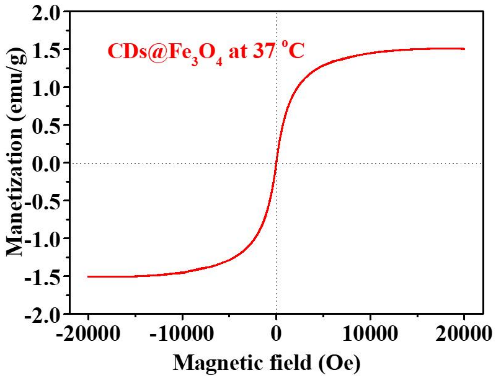

Figure S5 The magnetization (M-H) curves of CDs@Fe_3O_4 at 37 °C.

The VSM result at 37 °C suggests the CDs@Fe_3O_4 still maintain a certain degree of paramagnetism at physiological temperatures for PTT.

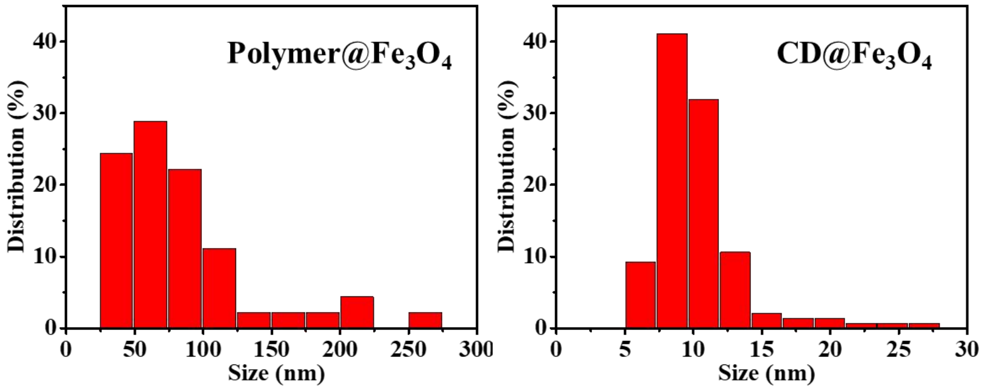

Figure S6 The size distribution histograms from TEM.

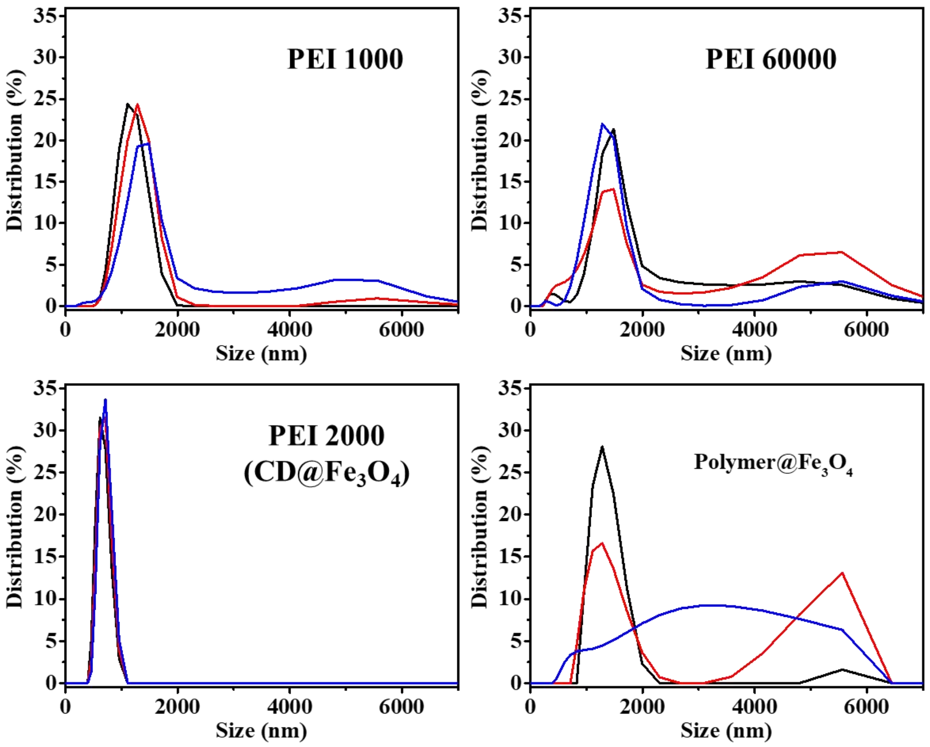

Figure S7 The size distribution from DLS.

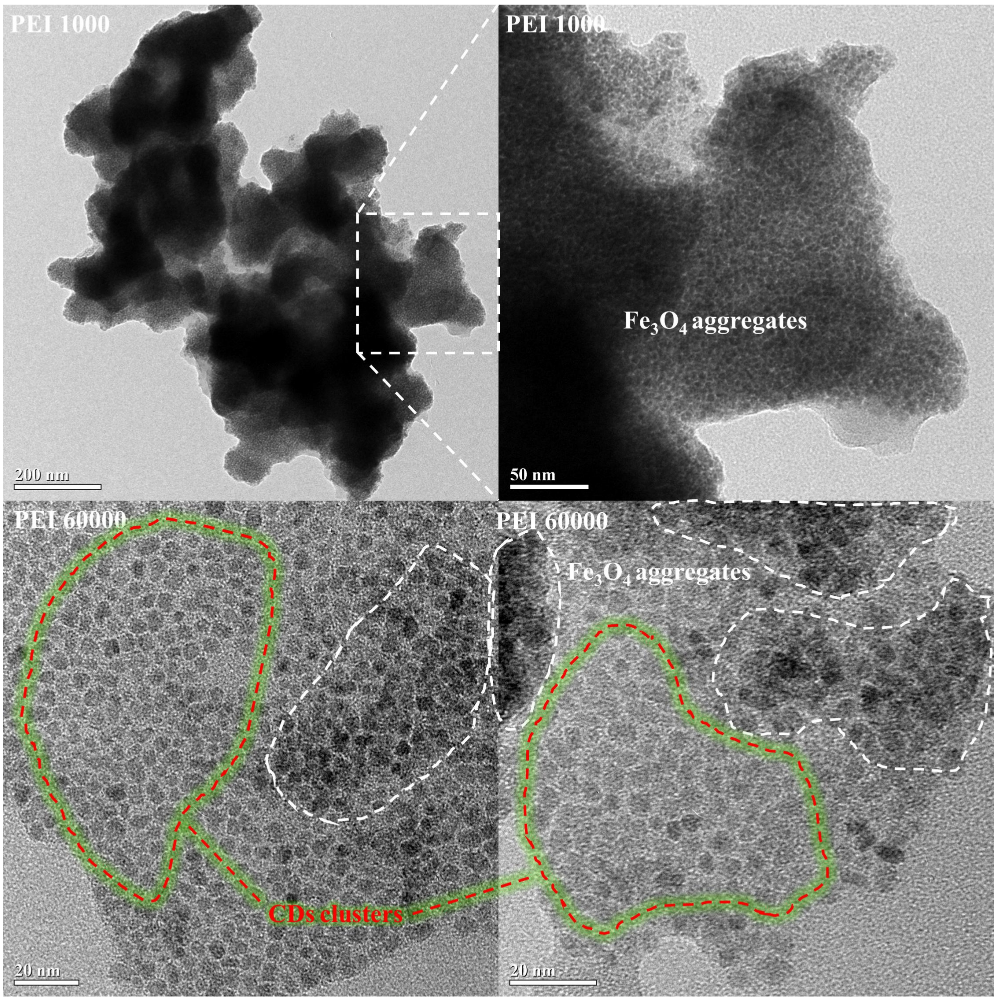

Figure S8 The TEM images of CDs@Fe_3O_4 with different weights of PEI.

From the TEM and DLS results, it can be seen that, The sample with 1000 Da of PEI, due to its small molecular weight, is not sufficient to crosslink CDs and Fe_3O_4, resulting in severe aggregation of Fe_3O_4 itself and hindering the generation of CDs. When the PEI molecular weight is 60000 Da, due to excessive molecular weight, one PEI molecule may crosslink multiple Fe_3O_4 and CDs, resulting in a large overall size of the aggregates. The CDs and Fe_3O_4 cannot be uniformly dispersed, which is not conducive to the stable existence of the nanocomposite. Only when the molecular weight reaches 2000 Da does the structure of Fe_3O_4-encapsulating CDs become stable, thereby ensuring that the overall dispersion and size meet the requirements for PTT.

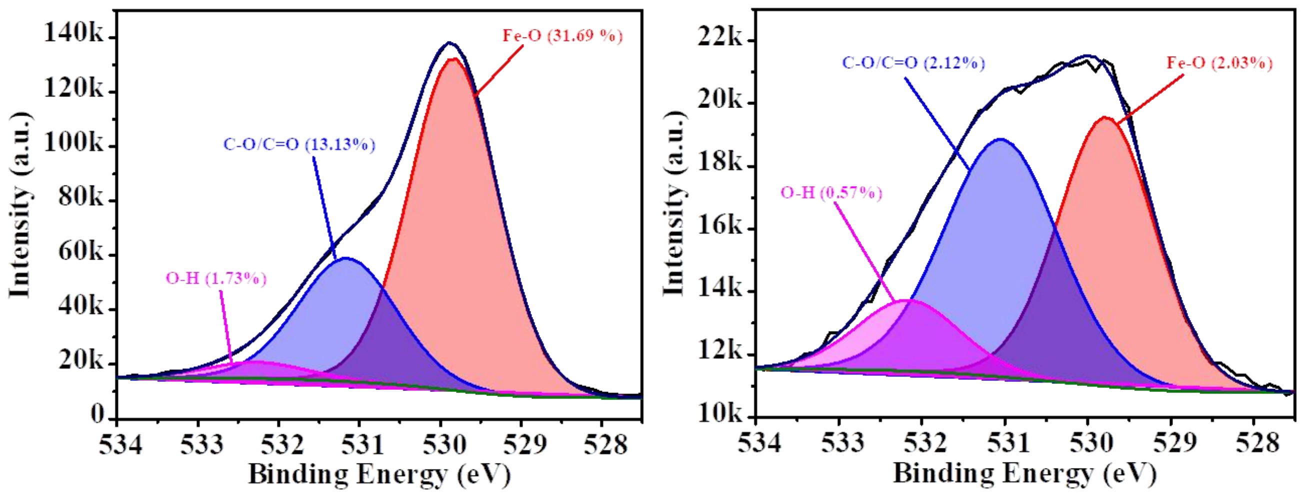

Figure S9. The high-resolution XPS of O 1s spectra of polymer@Fe_3O_4 (left) and CDs@Fe_3O_4 (right).

Table S1 The element content of polymer@Fe_3O_4 and CDs@Fe_3O_4 from XPS results.

	%	%	C-C %	π→π* %	%	N-C %	Satellite peak, %	Fe^{3+} %	Fe^{2+} %
Polymer@Fe_3O_4	4.67 (C=O)	12.68 (C-O/C-N)	7.6	0.2	1.01 (N-H)	4.56	5.64	10.88	6.21
CDs@Fe_3O_4	3.93 (C=N)	52.19 (sp^2 C)	26.01	0.5	0.69 (N=C)	10.65	0.19	0.43	0.56

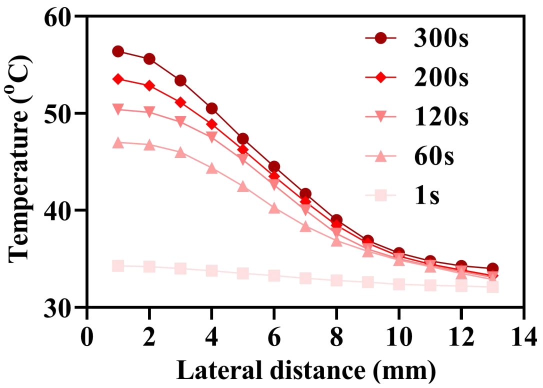

Figure S10 The temperature distribution curves versus the distances from the tumor center.

We extract the temperature data from the photothermal imaging and draw the temperature distribution curves versus distances from the tumor center. This result indicates that the high-temperature area generated by photothermal effect almost only exists in the tumor sized area, and the temperature rise of normal tissues around the tumor is limited, confirming its safety.

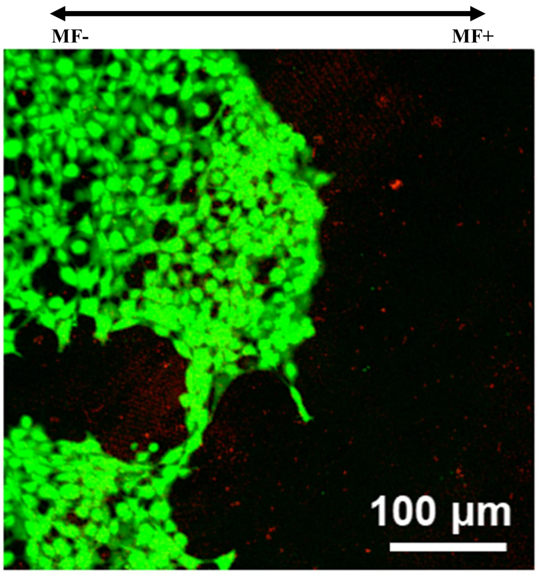

FigureS11 The Calcein-AM/PI dual-staining assay for cells co-incubated with CDs@Fe_3O_4.

The calcein-AM/PI dual-staining assay was also employed to evaluate the effect of MF enhanced PTT on cell viability. The verification via Calcein-AM/PI dual-staining assay showed that viable cells emitted specific green fluorescence, while dead cells were stained red by PI after lase irradiation. This further confirmed the regional specificity of the material's photothermal therapy under magnetic field guidance.

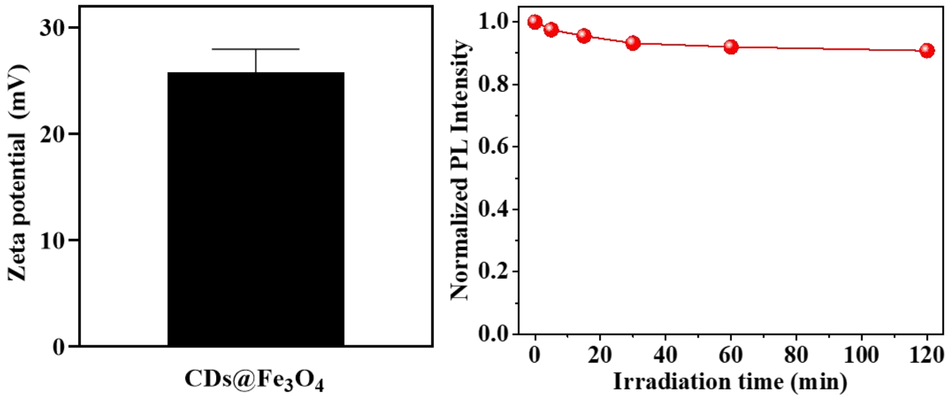

Figure S12 The zeta potential and PL stability with continuous light irradiation of the CDs@Fe_3O_4.

The zeta potential of the CDs@Fe_3O_4 was measured as 25.8 mV, indicating relatively good colloidal stability in aqueous dispersions. The PL intensity of CDs@Fe_3O_4 remained at 90% after continuous light irradiation for 2 hours, which confirms their excellent photostability. This ensure their stability performances in subsequent PTT.

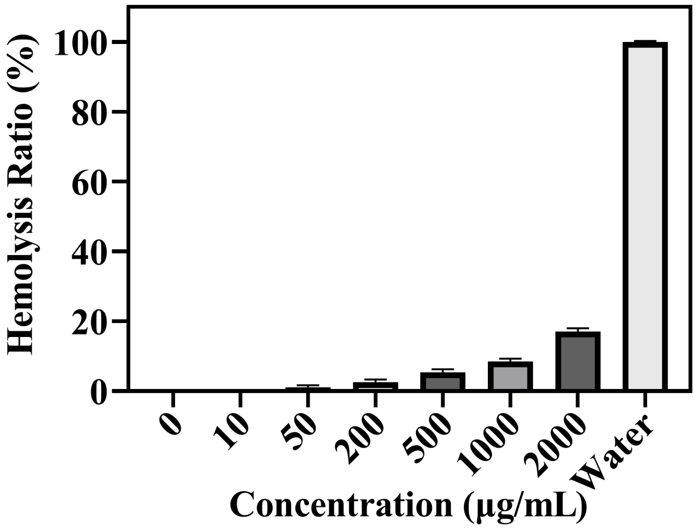

Figure S13 Hemolysis analysis results of CDs@Fe_3O_4. The blood in PBS and deionized water were used as the negative and positive controls, respectively.

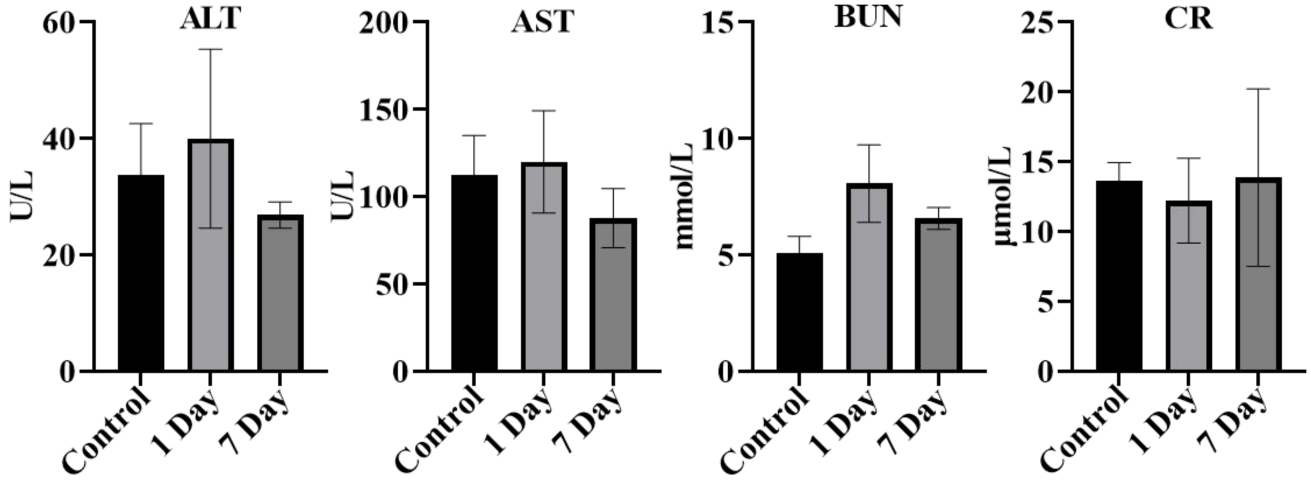

Figure S14 Biochemistry tests of the mice without and with CDs@Fe_3O_4 administration after 1 and 7 days. ALT (U/L): Alanine transaminase; AST (U/L): Aspartate aminotransferase; BUN (mmol/L): Blood urea nitrogen; CR (mmol L−1): Creatinine, n=4.

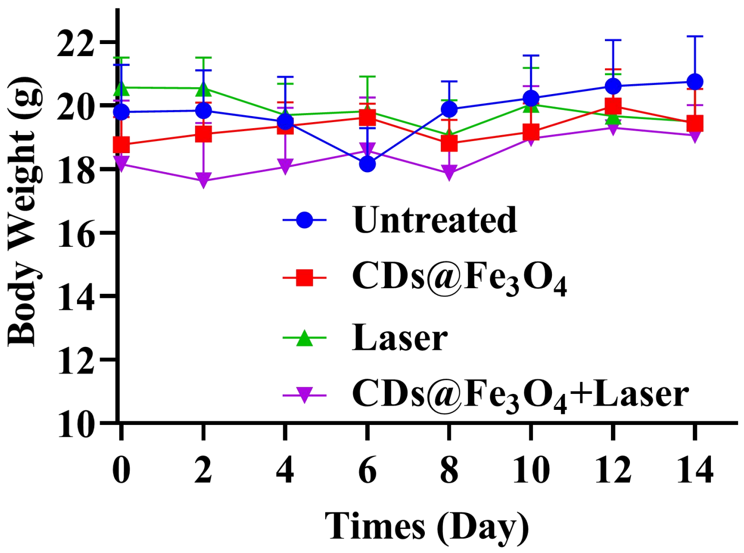

Figure S15 The body weight of different groups after PTT.

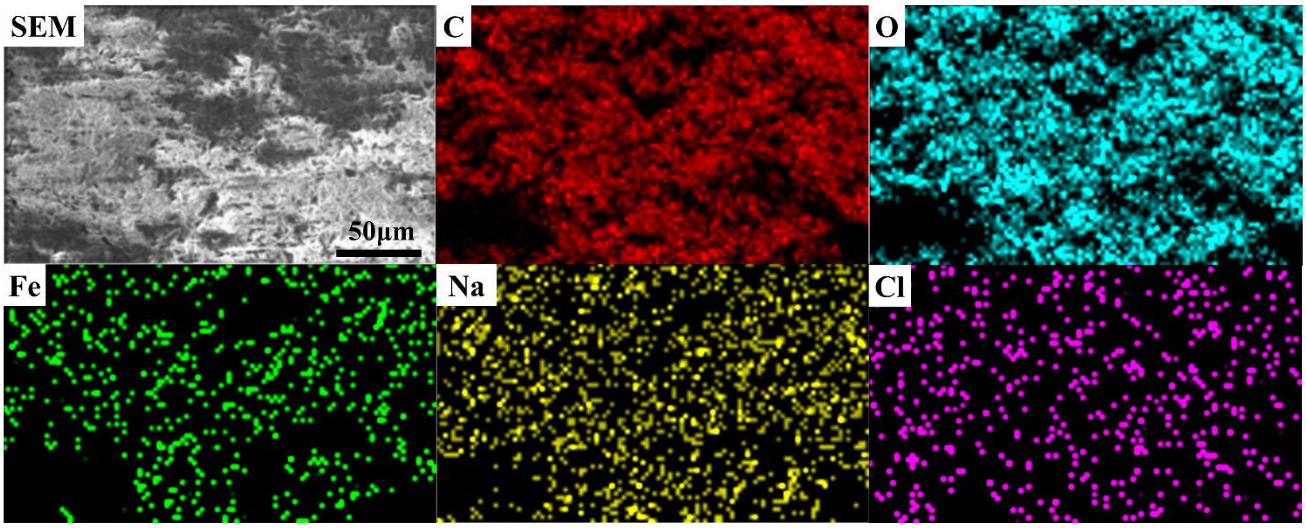

Figure S16. The SEM-EDS mapping of tumor tissue.

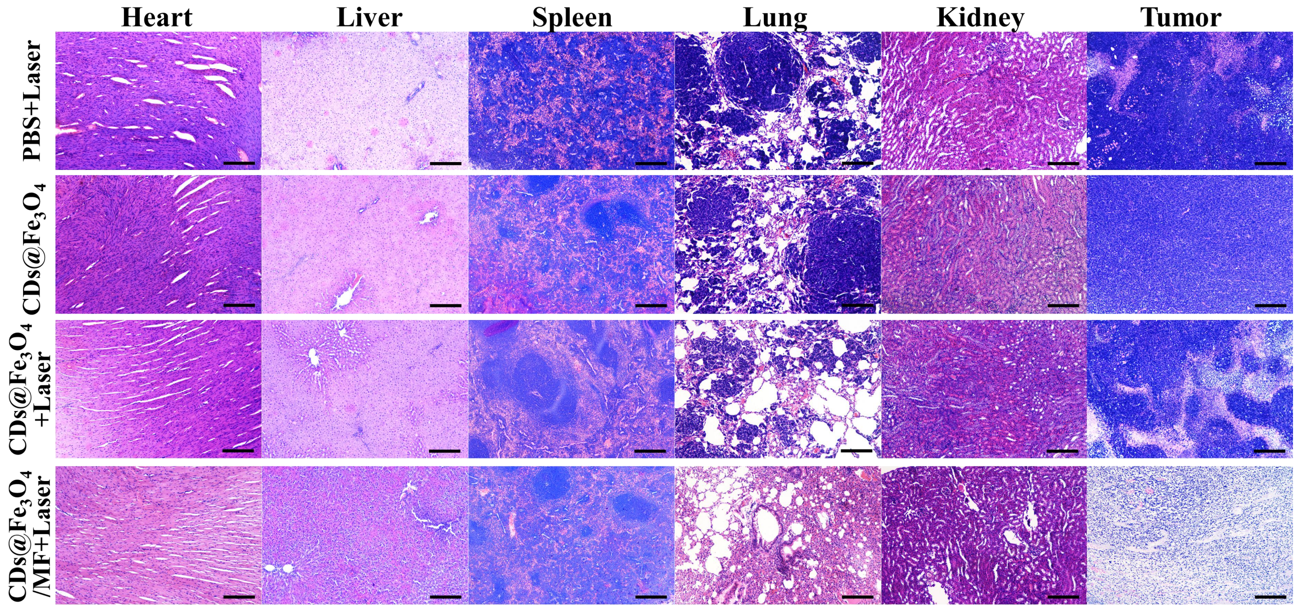

Figure S17. H&E staining of major organs and tumor tissues 30 days after PTT in different treatment groups of mice, scale bar: 200 μm.
